# Acute Effects of Intermittent and Continuous Static Stretching on Hip Flexion Angle in Athletes with Varying Flexibility Training Background

**DOI:** 10.3390/sports8030028

**Published:** 2020-03-03

**Authors:** Olyvia Donti, Vasiliki Gaspari, Kostantina Papia, Ioli Panidi, Anastasia Donti, Gregory C. Bogdanis

**Affiliations:** School of Physical Education & Sport Science, National and Kapodistrian University of Athens, 17237 Athens, Greece; vgaspari@phed.uoa.gr (V.G.); konpapia@phed.uoa.gr (K.P.); ipanidi@phed.uoa.gr (I.P.); adonti@phed.uoa.gr (A.D.)

**Keywords:** range of motion, hamstrings, stretching exercises, gymnastics, team sports, straight leg raise

## Abstract

Τhis study examined changes in hip joint flexion angle after an intermittent or a continuous static stretching protocol of equal total duration. Twenty-seven female subjects aged 19.9 ± 3.0 years (14 artistic and rhythmic gymnasts and 13 team sports athletes), performed 3 min of intermittent (6 × 30 s with 30 s rest) or continuous static stretching (3 min) of the hip extensors, with an intensity of 80–90 on a 100-point visual analogue scale. The order of stretching was randomized and counterbalanced, and each subject performed both conditions. Hip flexion angle was measured with the straight leg raise test for both legs after warm-up and immediately after stretching. Both stretching types equally increased hip flexion angle by ~6% (continuous: 140.9° ± 20.4° to 148.6° ± 18.8°, *p* = 0.047; intermittent: 141.8° ± 20.3° to 150.0° ± 18.8°, *p* = 0.029) in artistic and rhythmic gymnasts. In contrast, in team sports athletes, only intermittent stretching increased hip flexion angle by 13% (from 91.0° ± 7.2° to 102.4° ± 14.5°, *p* = 0.001), while continuous stretching did not affect hip angle (from 92.4° ± 6.9° vs. 93.1° ± 9.2°, *p* = 0.99). The different effect of intermittent vs. continuous stretching on hip flexion between gymnasts and team sports athletes suggests that responses to static stretching are dependent on stretching mode and participants training experience.

## 1. Introduction

Warm-up routines usually include static stretching in its simplest form [1]. Static stretching is a widely used type of flexibility training [2,3] which is used to increase joint range of motion (ROM) in a duration dependent manner [4]. In this type of stretching, a limb is moved near to its end point ROM and is typically maintained at this position for 15 to 60 s [5]. Transient increases in joint ROM following static stretching are due to a modified stretch sensation [6] and an increased compliance of the musculotendinous unit [7]. 

Loading characteristics of the stretching protocol, such as total stretch duration per muscle group, the duration of each stretching bout, rest between stretches, stretch intensity, and the muscle examined, influence acute joint ROM increases [8,9,10,11]. Previous research has reported that the total stretch duration is more important for joint ROM enhancement irrespective of whether stretching was performed in a continuous or intermittent manner [12,13]. In contrast, recent studies have found that different stretching modes (e.g., intermittent or continuous) induce dissimilar ROM changes [14,15]. Trajano, Nosaka, Seitz, and Blazevich, [14] compared an intermittent (5 × 1 min stretches with 15 s rest intervals between stretches) to a 5 min continuous stretching protocol, in young healthy adults and reported that only the continuous stretching protocol increased ankle angle during dorsiflexion, possibly due to a greater creep effect. In another study in elite male gymnasts, Bogdanis et al. [15] examined the effect of two different stretching protocols (3 × 30 s with 30 s rest, vs. 90 s) of the same total duration on hip and knee joint ROM. In that study it was found that intermittent and continuous stretching protocols induced similar increases in hip (+2.9° vs. +3.6°, *p* = 0.001, respectively) and knee joint ROM (+5.1° vs. +6.1°, *p* = 0.001, respectively) [15]. Such brief stretching durations (~30 s) are typically used in sports, since a typical warm-up routine includes 1–3 sets of shorter duration stretches (15–30 s) interspersed with rest intervals of equal duration, while the contra-lateral limb is being stretched [16,17]. Participants flexibility training experience may partly explain the discrepant results between studies. Along this line, Blazevich et al. [18] examined neuromuscular factors affecting maximum stretch limit and found that participants with a larger range of motion showed less resistance to stretch compared to less flexible participants due to a greater stretch tolerance. Cross-sectional studies comparing flexibility trained subjects (e.g., ballet dancers and rhythmic gymnasts) to controls or athletes from other sports also reported larger joint ROM in flexibility trained compared to untrained participants, as well as differences in muscle architecture [19,20]. 

Prolonged stretching durations (60–90 s or more) are used to maximize increases in ROM [4], facilitate recovery from injuries [21], prevent muscle mass loss in clinical conditions [22], and enhance performance in complex athletic tasks [23,24]. However, evidence is ambiguous regarding the effectiveness of performing static stretching in an intermittent or a continuous manner [14,15], especially when using stretching bouts lasting <60 s, as typically applied in sports. Also, the interaction of static stretching type and flexibility training background is largely unexplored. To this end, we aimed to examine changes in hip joint flexion angle after an intermittent (6 × 30 s with 30 s rest) or a continuous (180 s) static stretching protocol of equal total duration between athletes with different flexibility backgrounds. Hamstring muscles were chosen as they are important contributors to the work done at joints during explosive leg extensions [25], their extensibility determines hip and lumbar spine joint excursion [26], and a lack of hamstring flexibility is correlated to muscle injury [27]. It was hypothesized that flexibility trained athletes would respond differently to less-flexibility-trained counterparts, to an intermittent or continuous protocol of the same total duration.

## 2. Materials and Methods

### 2.1. Participants

Fourteen artistic and rhythmic gymnastics female college gymnasts were compared to thirteen female team sports athletes. All gymnasts trained 5–6 times per week (~180 min per training session) while team sports athletes competed in volleyball, basketball, and handball, and trained 2–3 times per week for 60–90 min. Gymnastics and team sports include a variety of locomotion activities using body weight, however gymnastics training additionally incorporates systematic daily flexibility training (~ 30–45 min), while team sports training includes less than 10 min of stretching exercises per training [28]. Participants anthropometric characteristics are shown in Table 1. Participants were healthy and did not report a lower limb injury for the past 6 months. 

### 2.2. Ethical Considerations

The study was approved by Institutional Ethics Committee (registration number: 1040, 14/02/2018). The design and conduct of the study was in accordance with the Helsinki declaration. Prior to the start of the study and after pertinent information of the procedures and potential risks involved were explained, participants signed a consent form. 

### 2.3. Experimental Design

Participants performed one familiarization and one main testing session. During the familiarization session, participants’ height and body mass were measured and they were familiarized with the testing procedures. The main testing session took place one week later and no intense exercise or stretching was allowed in the 48 h preceding testing. In order to stretch the hip extensors, the straight leg raise maneuver was performed to the point of discomfort, and it was applied on the one leg of the same individual in a continuous (180 s) and on the other leg in an intermittent manner (6 × 30 s with 30 s of rest in between). 

**Figure 1:**
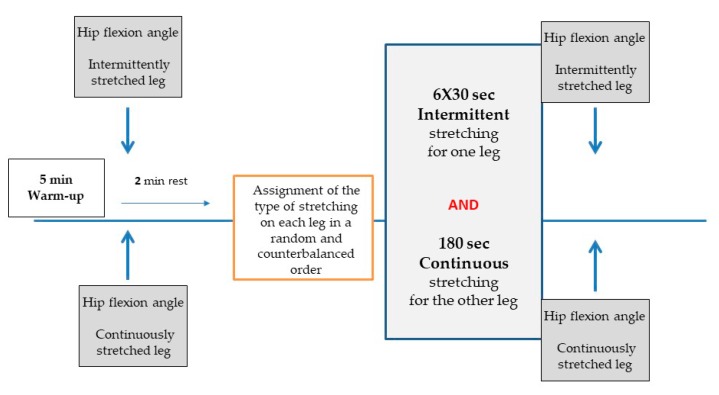
Schematic diagram of the study protocol.

In the main testing session, participants’ hip flexion angle, was assessed using the straight leg raise test, in two conditions: (a) immediately after warm-up, and (b) following stretching intervention. The warm-up included 5 min of jogging at a moderate intensity (50%–60% of maximal heart rate). During testing, participants performed with the one leg the intermittent protocol and with the other leg the continuous stretching protocol. The assignment of the stretching type and the order of legs was done in a random and counterbalanced order. A schematic representation of the study protocol is shown in Figure 1.

### 2.4. Static Stretching Procedure and Range of Motion Measurements

**Figure 2:**
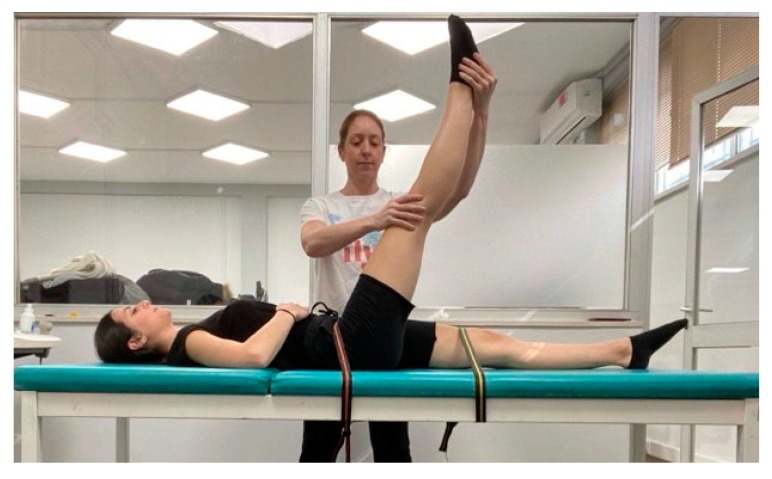
Straight leg raise movement.

The straight leg raise maneuver was chosen as a valid and reliable test of the extensibility of the hamstrings [29]. Furthermore, participants were familiar with this stretching movement because they used it in their training sessions. The static stretching protocols were applied and controlled by the same examiners. The straight leg raise was performed from a supine position on a physiotherapy bed, with the knee locked and the lower back flat on the bed (Figure 2). The lower back and the thigh of the non-stretched leg were stabilized with medical straps in order to prevent pelvic rotation. The participant’s head was not supported by pillow. The examiner grasped the participant’s heel of the tested leg with the one hand while with the other hand maintained the knee in an extended position. Slowly the examiner raised the stretched leg by flexing the hip. At the point of discomfort, the examiner maintained the stretch intensity for 30 s. The stretching movement was repeated for five more times, interspersed with 30 s of rest each time. During continuous stretching, the same procedure was followed and when the point of discomfort was reached, participants were instructed and verbally encouraged to maintain the stretch intensity for 3 min in order to induce the largest stretch they were willing to tolerate (Figure 2). 

Researchers supervised the position during testing, ensuring that both legs were straight, and the athletes kept a correct body alignment. Three anatomical markers were placed on hip (trochanterion), knee (femur–tibia joint line), and ankle (lateral malleolus) in order to analyze the images of hip flexion angle. A digital camera (Casio Exilim Pro EX-F1, Shibuya, Tokyo, Japan) placed perpendicular to the plane of motion of each leg, and aligned with the center of the hip joint, was used to record the position of the markers. Hip flexion angle was calculated using free software (Tracker 4.91 © 2016 Douglas Brown, Open Source Physics, Aptos, CA, USA). Straight leg raise angle was defined as the angle created by the intersection between horizontal and the line joining the hip, knee, and ankle markers. Intra-class correlation coefficients for hip angle was 0.96 (95% Confidence Intervals (CI): 0.81–0.99, *p* < 0.001). Participants gave feedback on stretch intensity to ensure that stretch achieved the point of discomfort (rating 80–90 to 100). Stretch intensity was indicated by the participants on a visual analogue scale used in previous studies, rated 0 (“no stretch discomfort at all”) to 100 (“maximal stretch discomfort”) [30]. 

### 2.5. Statistical Procedures

Descriptive statistics were calculated. Kolmogorov–Smirnov test checked for normality of data distribution. Unpaired *t*-test examined differences between groups in anthropometry. A three-way mixed model analysis of variance (ANOVA; time × stretching protocol × group) with time (rest vs. stretch) and stretching protocol (intermittent vs. continuous) as within-subjects factors and group (gymnasts vs. team sports athletes) as a between-subjects factor was conducted to examine the effect of stretching on hip flexion angle. Tukey’s post-hoc test was performed when a significant main or interaction effect was observed (*p* < 0.05). Effect sizes for pairwise comparisons were calculated by Cohen’s *d* [31]. Test–retest reliability was assessed by calculating the intra-class correlation coefficients (ICCs). Statistical significance was set at *p* < 0.05. Statistical analyzes were conducted using SPSS (SPSS Statistics Version 25.0, IBM corporation, Armonk, NY, USA).

## 3. Results

### Hip Flexion Angle

**Figure 3:**
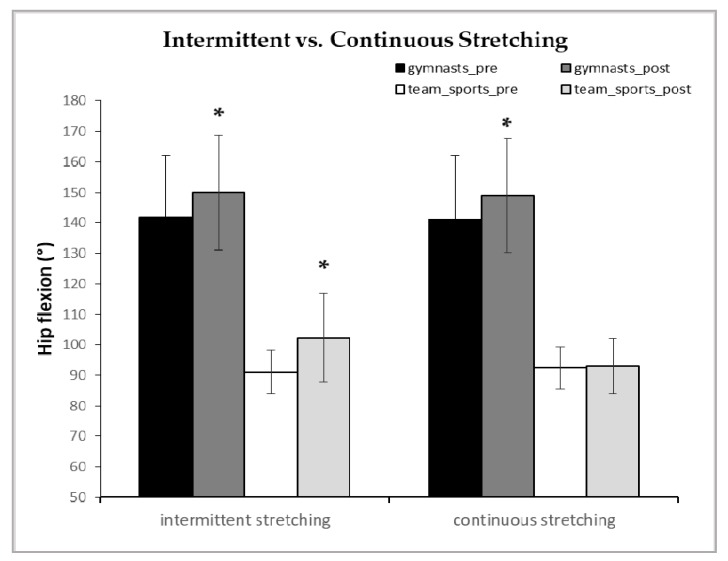
Hip flexion angle of gymnasts and team sport athletes during the intermittent and the continuous static stretching pre- and post-stretching intervention. Data are mean ± standard deviations of the mean. * *p* < 0.01 and *p* < 0.05 from pre-stretching.

There was a 3-way interaction of time x stretching protocol x group (*p* = 0.041, η^2^ = 0.157). As shown in Figure 3, the intermittent stretching protocol significantly increased hip flexion angle in gymnasts and team sports athletes compared to baseline, by 6% and 13% (+8.2° ± 6.3°, 95% CI = +5.5 to +11.0°, *p* = 0.029, Cohen’s *d* = 0.43 and +11.40° ± 16.3°, 95% CI = +2.6° to +20.2°, *p* = 0.001, Cohen’s *d* = 0.97, respectively). In contrast, the continuous stretching protocol resulted in an increase in hip flexion only in gymnasts by 6%, while no increase was observed in team sport athletes (+7.7° ± 5.5° *p* = 0.047, 95% CI= +5.2° to +10.2°, Cohen’s *d* = 0.41 vs, +1.0° ± 4.5°, *p* = 0.99, 95% CI = −1.1° to +3.1°, Cohen’s *d* = −0.09, respectively; see Figure 3).

## 4. Discussion

This study examined changes in hip joint flexion angle following an intermittent (6 × 30 s with 30 s rest) or a continuous (180 s) static stretching protocol of equal total duration between artistic and rhythmic gymnasts and team sports athletes. The main finding of this study was that both stretching protocols equally increased hip flexion angle in artistic and rhythmic female gymnasts while only intermittent stretching increased hip flexion angle in team sports athletes. 

Stretching is commonly performed in sports and rehabilitation to enhance joint range of motion [5,9], increase the distance over which muscle force is applied [32], and prevent muscle injuries [33]. Despite a considerable number of studies examining the acute effect of stretching on different performance measures (i.e., muscle force and power) [9,19] the characteristics of the stretching protocols to induce optimal joint ROM increases are not sufficiently documented. Some previous studies have shown that intermittent or cyclic stretching is highly effective in increasing joint ROM. [34,35,36]. In line with these findings, the results of this study also indicate that intermittent stretching (6 × 30 s) increases hip flexion angle in gymnasts and team sport athletes by 6% and 13%, respectively, thus this type of stretching may be used as an effective method to enhance ROM in athletic populations. In a previous study, Cipriani et al. [13] compared the effect of a 6 × 10 s vs. 2 × 30 s stretches repeated twice daily on hip flexion and found significant increases in joint ROM irrespective of stretching duration. In an older study, Roberts et al. [37] examined the effect of 5 × 9 s vs. 3 × 15 s of static stretching on passive ROM of the lower extremities. The authors observed similar increases in passive joint ROM following both stretching protocols [37]. Collectively, studies comparing intermittent stretching protocols with different durations of each stretching bout, but equal total duration, found ROM enhancement regardless of the duration of each stretching. To this end, it was suggested that after 10 slow stretches, the passive tension of the muscle–tendon unit was reduced for any given length of the tissue, thus indicating tissue relaxation [38]. Konrad et al. [35] also reported decreased muscle stiffness immediately after, and up to 5 min following, a 5 min intermittent stretch (5 × 60 s) in healthy individuals. The efficacy of intermittent stretching may be due to the effect of rest between stretches, which allows recovery of the nervous, muscular, and metabolic systems, so that muscles can continue to extend against a pulling load [34].

In contrast, continuous stretching may not always be as effective as intermittent stretching although there are reports for the contrary [14]. However, in this study, continuous stretching did not have any effect on hip flexion ROM in team sports athletes, despite the fact that total duration was similar between the two protocols. Nordez et al. [36] examined the effect of constant (180 s) vs. cyclic stretching (6 × 30 s) on passive torque–angle curve of the hamstring muscles and reported that different mechanisms were operating depending upon the type of stretching performed. Although the present study did not examine neurophysiological and mechanical factors underpinning ROM changes, it is possible that maintaining a stretch position for 180 s may result in an increased reflexive activation in less trained in flexibility individuals even though the participants were instructed to relax during stretching. The exact mechanisms of the reflex muscle activity are not known, although input from spinal and supraspinal regions might be influential [18]. In addition, stretching transiently decreases muscle–blood flow in proportion to the applied tensile force of the stretch [39,40]. Thus, an ischemic response can occur during a passive muscle stretch due to an increase in intramuscular pressure, which in turn, may interfere with muscle activation [39]. Trajano et al. [14] found a two-fold higher muscle ischemia in a continuous (5 min) compared to an intermittent (5 × 1 min) stretching protocol. In contrast, rest intervals between stretching, allowed for blood reperfusion and minimized the decrease in HbO_2_. 

The fact that gymnasts’ ROM increased equally with both stretching types may be due to their training background and the fact that prolonged static stretching is widely used in gymnastics. It is well established that flexibility training modifies stretch sensation (i.e., increases tolerance to the applied stretch) [41] so that individuals can tolerate higher levels of stretch for the same amount of perceived pain. Ιt has also been reported that ballet dancers and rhythmic gymnasts have longer fascicles at rest and during stretching compared to controls [19,20], and this may be related to lower muscle tone, muscle stiffness, and resistance to stretching compared with team sports athletes. A previous study that examined changes in hip and knee joint ROM, following an intermittent (3 × 30 s with 30 s rest) or a continuous (90 s) static stretching protocol also reported that both stretching protocols similarly increased hip extension and knee flexion in international level male gymnasts [15]. Furthermore, flexibility trained individuals may also suffer less blood flow decrease during static stretching. For example, Otsuki et al. [40] examined muscle oxygenation and fascicle length during passive stretching between ballet dancers and controls and reported that ballet dancers demonstrated greater muscle extensibility without a concomitant reduction in muscle–blood volume and muscle oxygenation. The authors concluded that increased muscle extensibility attenuates muscle-tone-related vessels compression and indices of muscle ischemia during stretching [40]. Thus, a lower level of ischemia during continuous stretching in gymnasts compared to team athletes may partially explain their different responses. However, it is noteworthy that in relative terms, i.e., as a percentage of the resting value, team sport athletes increased their hip flexion ROM following intermittent stretching by 13% vs. 6% compared with gymnasts, albeit the absolute hip angles reached were far greater in gymnasts (Figure 3). This may be due to a possible “ceiling effect” of flexibility in gymnasts, whose ROM could have reached an absolute maximum and was possibly restricted by factors other than muscle extensibility (e.g., articular structures). The fact that gymnasts had longer training experience and were lighter compared to team sports athletes is a limitation that should be acknowledged. However, gymnastics training, as well as somatotype and anthropometric demands are unique and different from other sports. 

## 5. Conclusions

In conclusion, both stretching types increased ROM in gymnasts, but only intermittent stretching was effective in team sports athletes. Thus, there is an interaction between stretching type and participants’ flexibility training background that should be considered among other factors (e.g., training experience, somatotype) when examining the acute effects of static stretching on range of motion enhancement. 

## Figures and Tables

**Table 1:** Anthropometric characteristics of the participants (means ± standard deviation).

Characteristics	Artistic and Rhythmic Gymnasts (*n* = 14)	Team Sports Athletes (*n* = 13)	*p*
Age (y)	20.64 ± 2.68	19.15 ± 3.29	0.207
Training experience (y)	11.64 ± 2.34	7.23 ± 4.66	0.004
Height (m)	166.64 ± 5.29	168.77 ± 6.53	0.364
Body mass (kg)	55.21 ± 4.59	59.77 ± 4.90	0.020
Body Mass Index (kg/m^2^)	19.86 ± 1.04	20.99 ± 1.46	0.031
